# Resting‐State EEG Spectral Power in Young Adults With Generalized Anxiety Disorder and Self‐Reported Habitual Short Sleep: A Case‐Control Study

**DOI:** 10.1002/brb3.71906

**Published:** 2026-11-06

**Authors:** Mengyu Wang, Lihua Wu, Ce Shi, Jing Gao, Mengyao Zhang, Haishui Jiang, Yongqi Yuan, Linze Wu, Huihui Yin, Yuan Gao, Wen Fu, Xinwang Chen

**Affiliations:** ^1^ School of Rehabilitation Sciences Henan University of Chinese Medicine Zhengzhou China; ^2^ The First Clinical Medical College of Anhui University of Chinese Medicine Hefei China; ^3^ The First Affiliated Hospital of Henan University of Chinese Medicine Zhengzhou China; ^4^ College of Acupuncture, Moxibustion and Tuina Henan University of Chinese Medicine Zhengzhou China

**Keywords:** generalized anxiety disorder, habitual short sleep, resting‐state EEG, spectral power, young adults

## Abstract

**Background:**

Generalized anxiety disorder (GAD) and insufficient sleep frequently co‐occur during early adulthood, yet resting‐state electroencephalographic (EEG) evidence in this population remains limited. The present study aimed to characterize resting‐state EEG spectral power in young adults with GAD who also reported habitual short sleep, compared with healthy controls.

**Methods:**

This exploratory case‐control study included 56 university students: 28 participants with GAD and self‐reported habitual short sleep and 28 healthy controls. Resting‐state EEG spectral power was quantified in the delta, theta, alpha, and beta bands. We retained unadjusted group comparisons for description and fitted age‐ and sex‐adjusted linear models for the primary inferential interpretation. Channel‐wise models likewise included group, age, and sex, with Benjamini–Hochberg FDR correction across the 30 channels within each frequency band.

**Results:**

After adjustment for age and sex, group coefficients remained positive for delta (2.51, 95% CI: 0.20 to 4.82), theta (2.52, 95% CI: 0.29 to 4.74), alpha (2.46, 95% CI: –0.12 to 5.03), and beta power (2.14, 95% CI: 0.13 to 4.15), but none met the FDR threshold (adjusted FDR *p* = 0.0501, 0.0501, 0.0608, and 0.0501, respectively). In unadjusted analyses, delta, theta, and beta differences survived FDR correction (FDR *p* = 0.0480 for each), whereas alpha did not (FDR *p* = 0.0553). In adjusted channel‐wise analyses, 26 channel‐by‐band comparisons across 15 channels survived within‐band FDR correction; all occurred in delta or theta and were concentrated over posterior and centroparietal electrodes.

**Conclusions:**

Whole‐scalp estimates were directionally higher in the GAD plus habitual short‐sleep group, but no age‐ and sex‐adjusted frequency‐band comparison survived FDR correction. Adjusted exploratory channel‐wise findings indicated a posterior and centroparietal concentration in delta and theta power. These estimates remain preliminary and do not permit attribution to GAD, habitual short sleep, or their interaction.

## Graphical abstract

Resting‐state EEG was compared between 28 university students with generalized anxiety disorder and self‐reported habitual short sleep and 28 healthy controls. Age‐ and sex‐adjusted whole‐scalp power estimates were higher in the GAD group across four frequency bands, but none met the false discovery rate threshold. Exploratory channel‐wise analyses identified higher posterior and centroparietal delta and theta power, with 26 channel‐by‐band comparisons across 15 channels surviving within‐band correction. The findings characterize an exploratory spatial pattern of low‐frequency EEG power in this combined clinical and sleep phenotype.

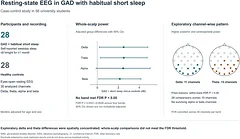

## 1 Introduction

Generalized anxiety disorder is characterized by persistent and excessive worry accompanied by somatic tension, autonomic arousal, and subjective distress (Teed et al. 2022). In early adulthood, the disorder coincides with ongoing neurocognitive maturation, heightened academic and social demands, and considerable instability in sleep‐wake schedules (Goodwin et al. 2017; Tsypes et al. 2013; Vytal et al. 2016; Zainal and Newman 2018). Habitual short sleep is likewise prevalent among university students, and naturally occurring short sleep may compound anxiety‐related daytime dysfunction (Meers et al. 2020; Yin et al. 2017).

Although anxiety and insufficient sleep frequently co‐occur in young adults, most prior research has addressed generalized anxiety disorder in isolation or relied on experimental sleep‐deprivation paradigms (Abdellah et al. 2021; Aydin Guclu et al. 2022). These approaches do not capture the naturalistic pattern of anxiety co‐occurring with self‐reported habitual short sleep. A descriptive neurophysiological characterization of this combined presentation may therefore be useful, while recognizing that the individual contributions of GAD and short sleep cannot be disentangled in a two‐group design.

Prior EEG studies in social anxiety disorder have reported alterations at both spectral and network levels. A small resting‐state and social‐performance study examined the frontal theta‐beta ratio and frontal midline theta (Al‐Ezzi et al. 2020). Subsequent work used resting‐state effective‐connectivity features within the default mode network to characterize symptom severity, including deep‐learning classification and partial directed coherence analyses across canonical frequency bands (Al‐Ezzi et al. 2021a; Al‐Ezzi et al. 2021b). Together, these studies illustrate the sensitivity of EEG to anxiety‐related neural variation, while also showing substantial heterogeneity in populations, anxiety phenotypes, EEG measures, and analytical methods.

Resting‐state EEG provides a practical way to describe ongoing oscillatory activity without imposing task demands (Khoo et al. 2024; Racz et al. 2019; Wang et al. 2024). Spectral analysis yields interpretable whole‐scalp and regional measures of rhythmic activity across canonical frequency bands and therefore complements network‐level approaches in an initial characterization (Javed et al. 2019; Llinás et al. 2015). At the same time, resting‐state spectral power is descriptive; it does not by itself identify specific cognitive operations, emotional processes, or causal pathways (Gordon et al. 2018; Manippa et al. 2025). Evidence specifically concerning young adults with clinically assessed GAD plus habitual short sleep remains limited.

This case‐control study therefore aimed to characterize resting‐state EEG spectral power in young adults with generalized anxiety disorder and self‐reported habitual short sleep, compared with healthy controls. Whole‐scalp mean band power was prespecified as the primary outcome, with channel‐wise topographic analyses treated as exploratory. Rather than inferring a condition‐specific explanation, our goal was to describe the scalp‐level spectral profile of this sample and provide a methodologically cautious basis for future factorial or longitudinal designs.

## 2 Materials and Methods

### 2.1 Study Design and Participants

This was a case‐control study conducted in university students recruited from the Longzihu and Dongming Road campuses of Henan University of Chinese Medicine. All participants provided written informed consent before enrollment. The protocol was approved by the Ethics Committee of the Third Affiliated Hospital of Henan University of Chinese Medicine (Ethics Approval No. 2023SH‐147; Renewal No. 2024–038).

Participants first completed the 7‐item Generalized Anxiety Disorder scale (GAD‐7) as a screening measure (Whittington and Borgogna 2025). Eligible participants then underwent face‐to‐face clinical evaluation by a trained clinical psychologist. The final analytic sample comprised 56 individuals: 28 participants in the GAD plus habitual short‐sleep group and 28 healthy controls. The sample size was determined primarily by recruitment feasibility within the predefined study period. No formal a priori power calculation was used to establish a confirmatory sample size. Given the modest sample of 28 participants per group, the analyses were intended to provide preliminary effect estimates rather than definitive hypothesis testing.

Inclusion criteria for the GAD plus habitual short‐sleep group were: age in early adulthood; clinical assessment consistent with generalized anxiety disorder according to ICD‐10 diagnostic criteria; and a self‐reported usual weekday nocturnal sleep duration of ≤6 h/night, with this sleep pattern persisting for at least 1 month. Healthy controls had no history of psychiatric or neurological disease and were classified at recruitment as not meeting the full operational criterion for habitual short sleep. General exclusion criteria for both groups included major neurological illness, history of severe head injury, substance dependence, current psychotic symptoms, or inability to complete EEG acquisition.

### 2.2 Clinical and Sleep Assessment

GAD status was assessed through a face‐to‐face clinical assessment conducted by a clinical psychologist trained in the application of ICD‐10 diagnostic criteria. The assessment combined clinical interview information with scale‐based symptom evaluation, including the GAD‐7 and HAMA‐14. Healthy controls underwent a corresponding clinical evaluation and had no evidence of generalized anxiety disorder or clinically significant anxiety symptoms on interview.

Because this study targeted a naturalistic pattern of short sleep rather than experimentally induced sleep deprivation, sleep exposure was assessed by self‐report. Habitual short sleep was operationally defined as a self‐reported usual weekday nocturnal sleep duration of ≤6 h/night that had persisted for at least 1 month. This study‐specific criterion was used for participant classification and should not be interpreted as a formal diagnostic criterion for a sleep disorder.

Average weekday and weekend sleep durations were recorded separately. The sleep deprivation index (SDI) was calculated as average weekend sleep duration minus average weekday sleep duration, following the approach described by Perotta et al. (2021). In the final analytic sample, the observed SDI range was 0–5 h. Higher positive values indicated greater weekend sleep extension relative to weekday sleep and were interpreted as a descriptive indicator of weekend catch‐up sleep or potential weekday sleep debt rather than as a validated diagnostic measure of sleep deprivation. SDI was not used as the criterion for classifying habitual short sleep. In the ID‐linked EEG control sample, weekday sleep duration ranged from 6.0 to 12.0 h; four controls reported exactly 6.0 h, none reported 6.5 h, and none reported a value >6 to <7 h. A reliable participant‐level field documenting persistence of the reported duration was unavailable, so whether those four controls met the full duration‐plus‐persistence definition could not be determined retrospectively. Because the sleep assessment relied on self‐report rather than objective monitoring, it was vulnerable to recall bias and exposure misclassification. The protocol lacked actigraphy, polysomnography, prospective sleep diaries, and validated screening for insomnia, circadian rhythm sleep‐wake disorders, obstructive sleep apnea, or shift‐work exposure.

### 2.3 EEG Acquisition

Resting‐state EEG was continuously recorded at 1000 Hz using a 32‐channel Brain Products system (Brain Products GmbH, Germany). Electrode impedance was maintained below 5 kΩ. Participants were tested in a quiet, dimly lit room and were instructed to remain awake, keep their eyes open, fixate on the monitor, minimize body movement, and avoid deliberate task engagement during recording. These instructions followed published recommendations for resting‐state EEG acquisition.

Participants were asked to avoid alcohol, caffeinated beverages, tea, and strenuous exercise for 24 h before testing. During acquisition, staff monitored the recording in real time and paused the procedure when obvious movement or drowsiness was detected.

### 2.4 EEG Preprocessing and Spectral Analysis

Raw resting‐state EEG data were imported from Brain Products files into EEGLAB. Channel locations were assigned using the standard 10–5 montage. Continuous data were band‐pass filtered at 0.5–30 Hz and notch filtered at 48–52 Hz to attenuate line noise. The data were then resampled to 250 Hz. A reference channel was temporarily restored during preprocessing, non‐target electrodes were removed to obtain the final analysis montage, and the data were re‐referenced to the bilateral mastoids (TP9/TP10). Continuous EEG was segmented into consecutive 2‐s epochs without baseline correction.

After initial preprocessing, noisy segments and bad channels were handled during an intermediate cleaning step, and bad channels were interpolated before independent component analysis (ICA). ICA was then performed using the extended Infomax algorithm with rank‐aware dimensionality reduction according to the effective data rank after preprocessing. Artifactual independent components were identified during preprocessing with reference to ICLabel‐assisted classification and removed. In a final artifact‐rejection step, epochs containing residual amplitudes exceeding ±100 µV were excluded. The remaining cleaned epochs were used for spectral analysis.

Spectral analysis was performed on the cleaned 2‐s EEG epochs. For each subject, channel, and epoch, a Hanning window was applied and the power spectrum was estimated by a 512‐point fast Fourier transform. Power spectral density values were converted to decibel units [10 × log10(PSD)] and then averaged across epochs. Frequency bands were defined as delta (1–4 Hz), theta (4–8 Hz), alpha (8–13 Hz), and beta (13–30 Hz). For channel‐wise analyses, mean band power was calculated by averaging spectral values across all frequency bins within each predefined band. For whole‐scalp analyses, band power was further averaged across channels to obtain one mean value per subject for each frequency band.

### 2.5 Statistical Analysis

All analyses were conducted at the participant level. Baseline characteristics were compared using Welch's *t*‐test, the Mann–Whitney *U* test, or the chi‐square test, as appropriate. For whole‐scalp mean log‐transformed absolute spectral power, unadjusted group‐only linear models were retained as descriptive comparisons. Covariate‐adjusted linear regression models included group (GAD + HSS vs. HC), age in years, and sex; because the groups differed in age and showed sex imbalance, these adjusted estimates were given primary inferential emphasis. Both raw and Benjamini–Hochberg FDR‐corrected *p* values are reported across the four frequency‐band comparisons. Unadjusted mean differences, 95% confidence intervals, and Hedges' *g* were retained for comparability. Channel‐wise analyses were exploratory: a separate linear model, spectral power ∼ group + age + sex, was fitted for each channel and frequency band, and group *p* values were FDR‐corrected across the 30 channels separately within each band. Adjusted group coefficients, standard errors, 95% confidence intervals, raw *p* values, and within‐band FDR *p* values are reported. These covariate‐adjusted analyses were not prespecified and should be interpreted as a reviewer‐requested reanalysis.

## 3 Results

### 3.1 Participant Characteristics

The GAD plus habitual short‐sleep group was younger than the healthy‐control group, whereas sex distribution and education did not differ significantly (Table 1). Anxiety scores and self‐reported sleep measures differed in the expected directions. In the ID‐linked control records used for the EEG reanalysis, weekday sleep duration ranged from 6.0 to 12.0 h. Four controls reported exactly 6.0 h; no control reported 6.5 h or a value >6 to <7 h. Because a participant‐level duration‐persistence field was unavailable, it was not possible to determine retrospectively whether the four observations at 6.0 h met the full habitual short‐sleep definition.

**TABLE 1:** Baseline characteristics of the GAD plus habitual short‐sleep group and healthy controls.

Characteristic	GAD + HSS (*n* = 28)	HC (*n* = 28)	Statistical test	Statistic	*p* value	Effect size
Age (years)	19.0 [18.0, 21.0]	21.0 [20.0, 21.0]	Mann–Whitney *U*	*U* = 268.0; *Z* = −2.09	0.037	*r* = 0.28
Sex, male/female	7/21	13/15	Chi‐square test	χ^2^ = 2.80	0.094	—
Education (years)	14.61 ± 1.40	14.82 ± 0.94	Welch's *t*‐test	*t*(47.4) = −0.67	0.505	—
Weekday sleep duration (h)	6.0 [5.0, 6.0]	7.0 [6.8, 8.0]	Mann–Whitney *U*	*U* = 106.5; *Z* = −4.83	< 0.001	*r* = 0.65
Weekend sleep duration (h)	8.0 [7.0, 9.0]	9.0 [8.0, 10.0]	Mann–Whitney *U*	*U* = 263.5; *Z* = −2.15	0.032	*r* = 0.29
GAD‐7 score	11.0 [10.5, 13.0]	2.5 [1.0, 5.0]	Mann–Whitney *U*	*U* = 784.0; *Z* = 6.45	< 0.001	*r* = 0.86
HAMA‐14 score	22.0 [15.0, 27.0]	4.0 [1.0, 7.0]	Mann–Whitney *U*	*U* = 782.0; *Z* = 6.39	< 0.001	*r* = 0.85
Sleep deprivation index (SDI)	2.0 [1.0, 3.0]	1.5 [0.8, 2.0]	Mann–Whitney *U*	*U* = 530.0; *Z* = 2.33	0.020	*r* = 0.31

*Note*: Continuous variables are reported as mean ± SD or median [interquartile range] as appropriate. HSS, habitual short sleep; HC, healthy control; HAMA‐14, 14‐item Hamilton Anxiety Scale; SDI, sleep deprivation index, calculated as average weekend sleep duration minus average weekday sleep duration. The observed SDI range in the final analytic sample was 0–5 h. The separate ID‐linked audit of the EEG controls found a weekday sleep range of 6.0–12.0 h, including four observations at exactly 6.0 h.

### 3.2 Primary Outcome: Whole‐Scalp Spectral Power

The age‐ and sex‐adjusted analyses were given primary inferential emphasis. Adjusted group coefficients (GAD + HSS minus HC) were 2.51 for delta power (95% CI: 0.20 to 4.82; raw *p* = 0.0341), 2.52 for theta power (95% CI: 0.29 to 4.74; raw *p* = 0.0274), 2.46 for alpha power (95% CI: –0.12 to 5.03; raw *p* = 0.0608), and 2.14 for beta power (95% CI: 0.13 to 4.15; raw *p* = 0.0376). After FDR correction across the four bands, the corresponding *p* values were 0.0501, 0.0501, 0.0608, and 0.0501; therefore, no adjusted band‐level comparison met the *p* < 0.05 FDR threshold (Table 2). The estimates were compatible with directionally higher power but remained imprecise after accounting for demographic imbalance.

**TABLE 2:** Primary whole‐scalp comparisons of spectral power between groups.

Frequency band	Adjusted difference (95% CI)	Adjusted raw *p*	Adjusted FDR *p*	GAD + HSS mean ± SD	HC mean ± SD	Unadjusted difference (95% CI)	Hedges' *g*	Unadjusted raw *p*	Unadjusted FDR *p*
Delta (1–4 Hz)	2.51 (0.20 to 4.82)	0.0341	0.0501	5.32 ± 2.50	2.87 ± 5.29	2.45 (0.23 to 4.66)	0.58	0.0313	0.0480
Theta (4–8 Hz)	2.52 (0.29 to 4.74)	0.0274	0.0501	−0.96 ± 2.22	−3.44 ± 5.19	2.48 (0.34 to 4.62)	0.61	0.0238	0.0480
Alpha (8–13 Hz)	2.46 (–0.12 to 5.03)	0.0608	0.0608	−2.44 ± 2.63	−4.88 ± 6.04	2.44 (–0.06 to 4.93)	0.52	0.0553	0.0553
Beta (13–30 Hz)	2.14 (0.13 to 4.15)	0.0376	0.0501	−7.58 ± 2.15	−9.66 ± 4.63	2.07 (0.14 to 4.01)	0.57	0.0360	0.0480

*Note*: Mean values are log‐transformed absolute power spectral density values (10 × log10[PSD]). Mean differences and coefficients were calculated as GAD + HSS minus HC. Adjusted estimates came from linear models including group, age, and sex and are shown first because they form the primary inferential basis of the revision. FDR correction was applied across the four bands. An adjusted FDR *p* value of 0.0501 does not meet the *p* < 0.05 threshold. Unadjusted confidence intervals and Hedges' *g* are retained as descriptive effect‐size estimates.

In the retained unadjusted comparisons, the GAD plus habitual short‐sleep group had higher mean power across all four bands. Delta (mean difference = 2.45, 95% CI: 0.23 to 4.66; Hedges' *g* = 0.58), theta (2.48, 95% CI: 0.34 to 4.62; Hedges' *g* = 0.61), and beta (2.07, 95% CI: 0.14 to 4.01; Hedges' *g* = 0.57) survived FDR correction (FDR *p* = 0.0480 for each), whereas alpha did not (2.44, 95% CI: –0.06 to 4.93; Hedges' *g* = 0.52; FDR *p* = 0.0553). These unadjusted findings are presented descriptively and do not override the adjusted inference. Whole‐scalp spectra are shown in Figure 1.

**FIGURE 1:**
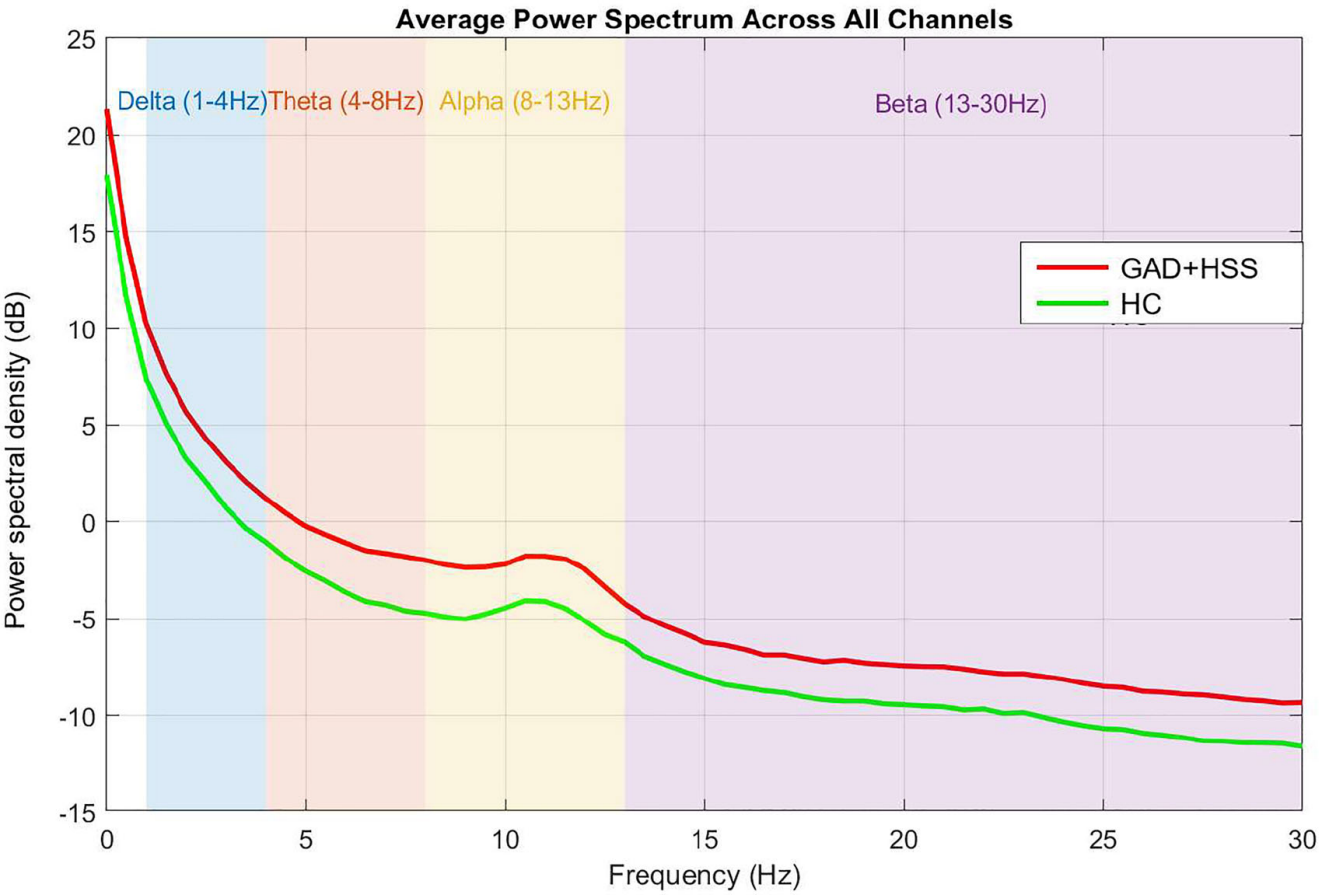
Average resting‐state log‐transformed EEG power spectral density across all channels in the GAD + HSS and HC groups. The GAD + HSS curve showed a descriptive upward shift relative to the HC curve across the canonical frequency range.

### 3.3 Exploratory Channel‐Wise Spectral Topography

Descriptive group topographies for the delta, theta, alpha, and beta bands are shown in Figure 2. Exploratory channel‐wise models included group, age, and sex and used within‐band FDR correction across 30 channels. Twenty‐six channel‐by‐band comparisons across 15 channels survived FDR correction. Delta‐band coefficients were significant at CP5, CP6, O1, O2, Oz, P4, P7, P8, Pz, PO9, and PO10. Theta‐band coefficients were significant at CP1, CP2, CP5, CP6, Cz, O1, O2, Oz, P4, P7, P8, Pz, T8, PO9, and PO10. All surviving coefficients were positive (GAD + HSS > HC), with FDR *p* values ranging from 0.0185 to 0.0499. No alpha‐ or beta‐band channel survived within‐band FDR correction. The adjusted findings therefore support an exploratory posterior and centroparietal concentration in delta and theta rather than a generalized channel‐level effect across all bands (Table 3; Figure 3).

**FIGURE 2:**
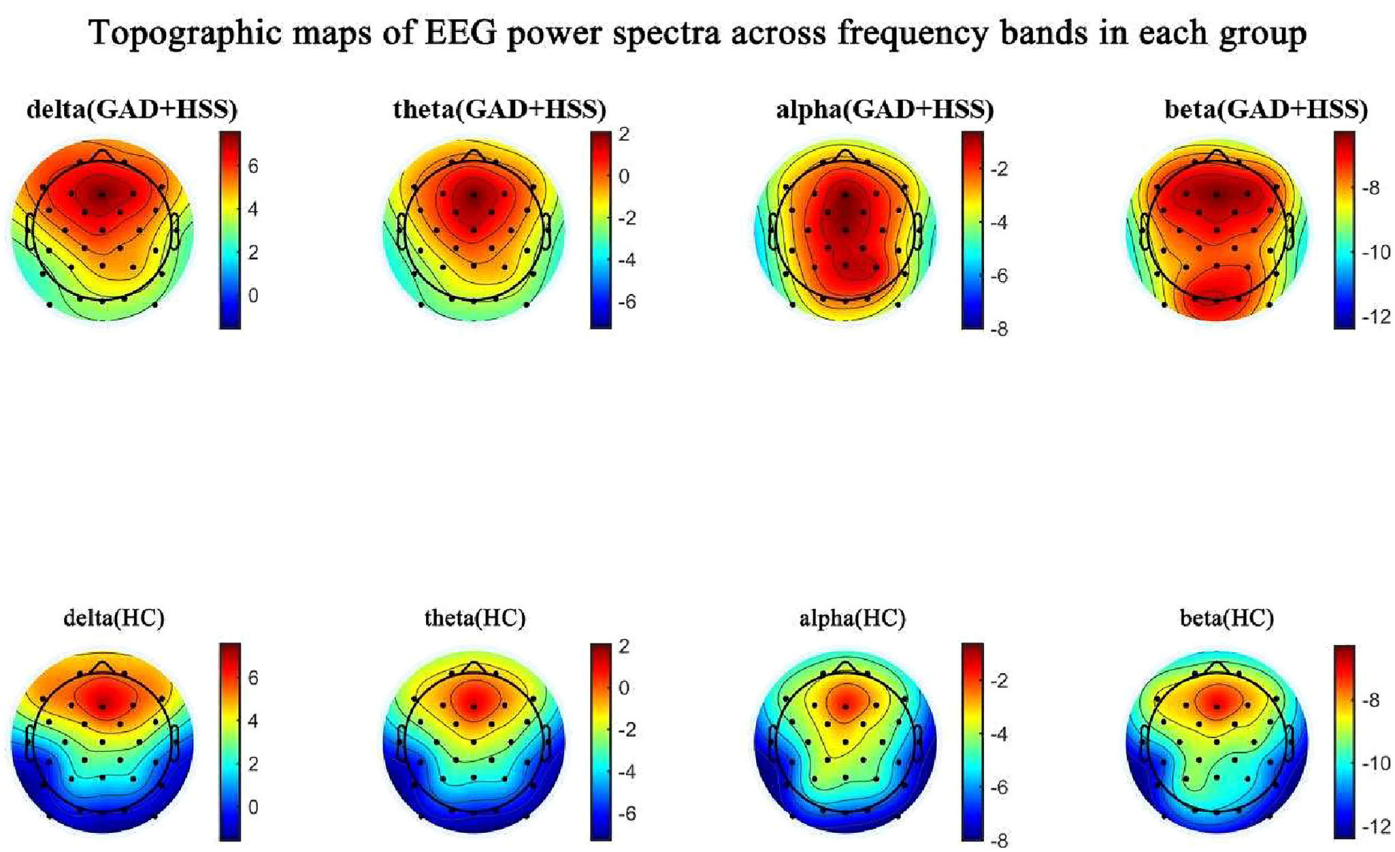
Descriptive topographic maps of EEG spectral power in the delta, theta, alpha, and beta bands for the GAD + HSS and HC groups. Warmer colors indicate higher spectral power. These unadjusted group maps are provided for visualization and are not inferential statistical maps.

**TABLE 3:** Summary of exploratory channel‐wise differences surviving FDR correction.

Channel	Significant adjusted bands	Direction	Minimum FDR *p*	Region
CP1	Theta	GAD + HSS > HC	0.0491	Centroparietal
CP2	Theta	GAD + HSS > HC	0.0491	Centroparietal
CP5	Delta, theta	GAD + HSS > HC	0.0185	Centroparietal
CP6	Delta, theta	GAD + HSS > HC	0.0199	Centroparietal
Cz	Theta	GAD + HSS > HC	0.0491	Central midline
O1	Delta, theta	GAD + HSS > HC	0.0199	Occipital
O2	Delta, theta	GAD + HSS > HC	0.0185	Occipital
Oz	Delta, theta	GAD + HSS > HC	0.0185	Occipital midline
P4	Delta, theta	GAD + HSS > HC	0.0426	Parietal
P7	Delta, theta	GAD + HSS > HC	0.0185	Parietal
P8	Delta, theta	GAD + HSS > HC	0.0185	Parietal
Pz	Delta, theta	GAD + HSS > HC	0.0322	Parietal midline
T8	Theta	GAD + HSS > HC	0.0280	Temporal
PO9	Delta, theta	GAD + HSS > HC	0.0185	Parieto‐occipital
PO10	Delta, theta	GAD + HSS > HC	0.0185	Parieto‐occipital

*Note*: Only channels showing at least one FDR‐significant adjusted group coefficient are listed. All listed coefficients were positive (GAD + HSS > HC). Models included group, age, and sex; Benjamini–Hochberg FDR correction was applied separately across 30 channels within each frequency band. No alpha‐ or beta‐band channel survived FDR correction.

**FIGURE 3:**
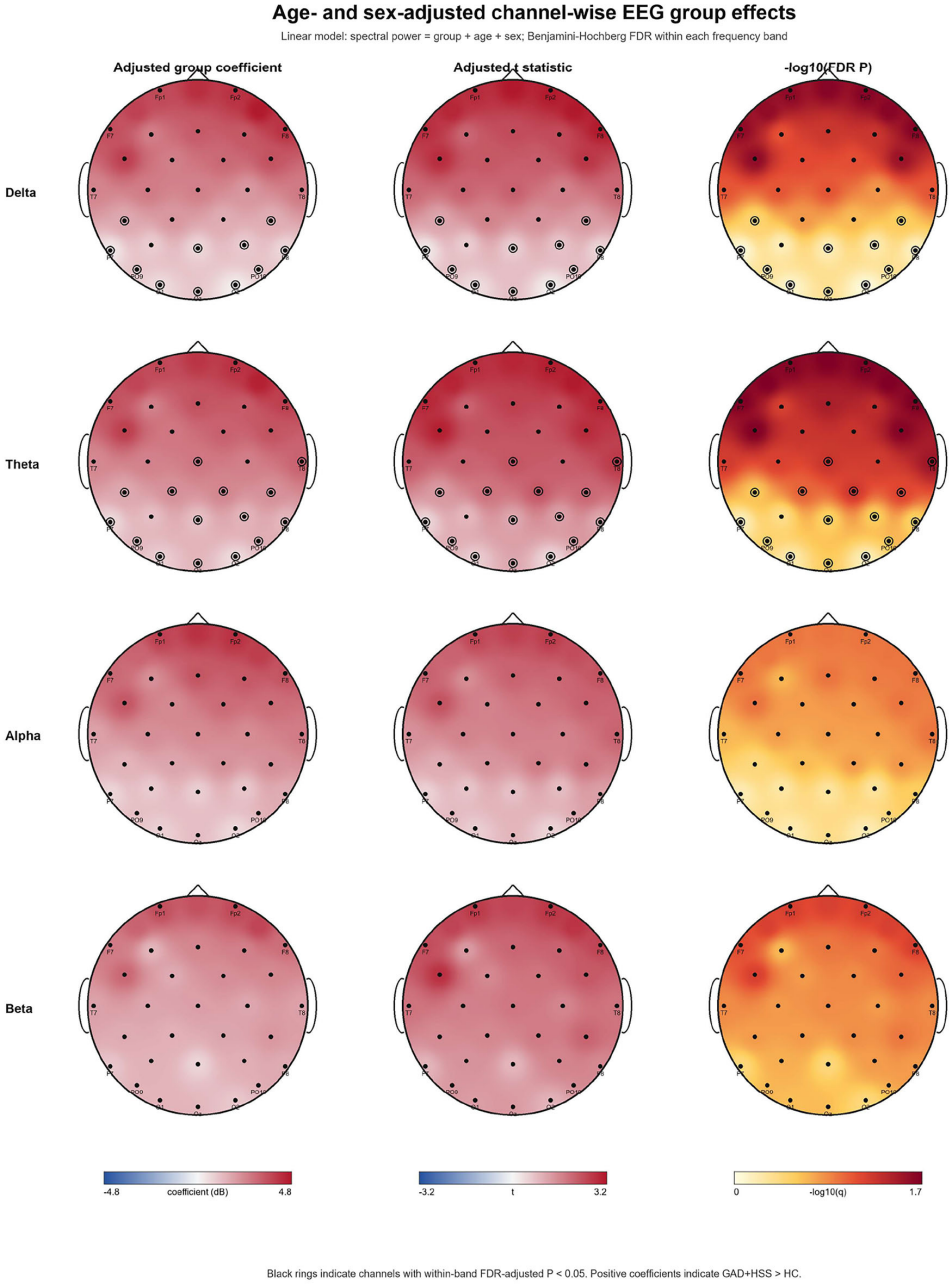
Statistical topographic maps from exploratory age‐ and sex‐adjusted linear models of channel‐wise EEG spectral power. For each band, maps show the adjusted group coefficient (GAD + HSS minus HC), adjusted *t* statistic, and –log10(within‐band FDR *p*). Black rings denote channels with FDR *p* < 0.05. Positive coefficients indicate higher power in the GAD + HSS group.

After age and sex adjustment, statistically supported channel‐wise differences were concentrated over posterior and centroparietal electrodes in the delta and theta bands. In the absence of source reconstruction or connectivity analysis, these findings are best interpreted as exploratory scalp‐level spatial patterns.

## 4 Discussion

The principal whole‐scalp finding was that age‐ and sex‐adjusted group coefficients remained positive across delta, theta, alpha, and beta power, but none survived FDR correction. Delta, theta, and beta estimates had raw *p* values below 0.05, yet their adjusted FDR *p* values were 0.0501, immediately above the 0.05 threshold. The unadjusted delta, theta, and beta comparisons survived FDR correction, but these descriptive results should not be interpreted as robust group differences independent of demographic imbalance. At the exploratory channel level, adjusted delta and theta effects were concentrated over posterior and centroparietal electrodes, whereas no alpha‐ or beta‐band channel survived within‐band FDR correction.

Given the two‐group design, the findings are best regarded as preliminary estimates rather than evidence of an independent disease‐specific EEG pattern. The adjusted whole‐scalp confidence intervals were wide, and *p* values close to the multiplicity threshold are vulnerable to modest changes in model specification or sample composition. The adjusted posterior delta/theta channel pattern provides a spatially focused hypothesis for replication, but scalp spectral power alone does not warrant inferences about vigilance, cortical excitability, emotion regulation, or causal circuitry (Greer et al. 2021; Syan et al. 2021). Causal attribution would require a factorial design separating GAD with normal sleep, GAD with short sleep, short sleep without GAD, and healthy controls.

The observed spatial pattern can be considered alongside prior social‐anxiety EEG studies that examined frontal spectral ratios and default‐mode effective connectivity (Al‐Ezzi et al. 2020, 2021a, Al‐Ezzi et al. 2021b). Those studies used different anxiety phenotypes and analytical targets, so they do not establish a common biomarker or directly validate the present power findings. Instead, they illustrate that anxiety‐related EEG variation may be expressed through multiple spectral and network measures.

Several limitations should be noted. First, the two‐group design cannot distinguish effects attributable to GAD, habitual short sleep, their interaction, or other group differences. Second, age and sex imbalance may introduce residual confounding. Although adjusted coefficients were positive, no whole‐scalp band survived FDR correction, and the modest sample limited precision. Third, sleep duration was retrospectively self‐reported. The ID‐linked control audit found four observations at exactly 6.0 h, while the participant‐level persistence field needed to evaluate the full habitual short‐sleep definition was unavailable; exposure misclassification therefore cannot be excluded. The protocol also lacked actigraphy, polysomnography, prospective sleep diaries, and validated screening for insomnia, circadian rhythm disorders, obstructive sleep apnea, or shift‐work exposure (Gabelle et al. 2017; Miner et al. 2022; Ruppert and Kilic‐Huck 2018; Scarlett et al. 2021). Fourth, SDI was a descriptive index of weekend sleep extension and was not a diagnostic measure. Fifth, channel‐wise models were exploratory and involved multiple comparisons; the posterior delta/theta pattern requires independent replication. Sixth, detailed component‐class and epoch‐level artifact summaries were not fully preserved. Finally, the analysis remained at the scalp level without source localization, connectivity analysis, or behavioral correlates (Leone et al. 2024).

Future work should adopt a four‐group factorial design (GAD with normal sleep, GAD with short sleep, short sleep without GAD, and healthy controls) alongside longitudinal studies, objective sleep measurement, larger samples, source‐localization, and functional‐connectivity analyses. Additional details are provided in Supplementary Table S1.

## 5 Conclusion

Age‐ and sex‐adjusted whole‐scalp coefficients were directionally higher in young adults with GAD and self‐reported habitual short sleep than in healthy controls, but no frequency‐band comparison survived FDR correction. Adjusted exploratory channel‐wise models identified a posterior and centroparietal concentration of delta and theta differences, with no surviving alpha or beta channel. The estimates are preliminary, sensitive to the limitations of a modest two‐group sample, and do not permit attribution to GAD, habitual short sleep, or their interaction. Replication in larger factorial and longitudinal studies with objective sleep measurement is required.

## Author Contributions

**Mengyu Wang** and **Lihua Wu** designed the study and drafted the manuscript. **Ce Shi**, **Jing Gao**, **Mengyao Zhang**, **Haishui Jiang**, **Yongqi Yuan**, **Linze Wu**, **Huihui Yin**, and **Yuan Gao** participated in the experiments. **Wen Fu** and **Xinwang Chen** analyzed the data. All authors read and approved the final manuscript.

## Funding

This work was supported by the National Natural Science Foundation of China (82305417); Construction Project of Chinese Medicine in Henan Province (HSRP‐DFCTCM‐2023‐7‐15); Science and Technology Research Project of Henan Province (232102311217); and the Henan Province Science and Technology Research and Development Joint Fund (242301420089).

## Conflicts of Interest

The authors declare that the research was conducted in the absence of any commercial or financial relationships that could be construed as a potential conflicts of interest.

## Supporting information




**Supplementary Table S1**: brb371906‐sup‐0001‐TableS1.docx

## Data Availability

The data underlying this study are available from the corresponding authors upon reasonable request.
