# Supplementary material for: Resting‐State EEG Spectral Power in Young Adults With Generalized Anxiety Disorder and Self‐Reported Habitual Short Sleep: A Case‐Control Study
Source: Brain Behav. 2026 Nov 6;16(11):e71906. doi: 10.1002/brb3.71906 (PMC13741991; doi:10.1002/brb3.71906)
Supplement: Supplementary file 1 [file BRB3-16-e71906-s001.docx]

Supplementary Table S1

**Sensitivity analysis excluding controls reporting weekday sleep duration ≤6 h/night**

| **Band** | **Main adjusted difference** | **Main 95% CI** | **Main raw P** | **Main FDR P** | **Sensitivity adjusted difference** | **Sensitivity 95% CI** | **Sensitivity raw P** | **Sensitivity FDR P** |
| --- | --- | --- | --- | --- | --- | --- | --- | --- |
| Delta (1–4 Hz) | 1.61 | −0.67 to 3.89 | 0.163 | 0.163 | 2.29 | −0.37 to 4.95 | 0.090 | 0.090 |
| Theta (4–8 Hz) | 1.87 | −0.36 to 4.11 | 0.099 | 0.163 | 2.67 | 0.10 to 5.25 | 0.042 | 0.059 |
| Alpha (8–13 Hz) | 2.08 | −0.62 to 4.77 | 0.128 | 0.163 | 3.10 | 0.08 to 6.11 | 0.044 | 0.059 |
| Beta (13–30 Hz) | 1.57 | −0.48 to 3.61 | 0.131 | 0.163 | 2.53 | 0.24 to 4.82 | 0.031 | 0.059 |

**Notes:** Differences are GAD+HSS minus HC from linear regression models including group, age, and sex. The main analysis included 28 GAD+HSS participants and 28 controls. The sensitivity analysis included 28 GAD+HSS participants and 21 controls after excluding seven controls who reported weekday sleep duration of ≤6 h/night and lacked retained participant-level documentation of persistence for at least 1 month. FDR P values were calculated across the four frequency bands within each analysis.
